# Anti-Sp4 and anti-CCAR1 autoantibodies in UK *vs* US patients with adult and juvenile-onset anti-TIF1γ-positive myositis

**DOI:** 10.1093/rheumatology/keae574

**Published:** 2024-11-07

**Authors:** Fionnuala K McMorrow, Lucy R Wedderburn, Hector Chinoy, Alexander Oldroyd, Janine A Lamb, Lisa G Rider, Andrew L Mammen, Livia Casciola-Rosen, Neil J McHugh, Sarah L Tansley, UK JDCBS and Childhood Myositis Heterogeneity Study Group, Kate Armon, Louise Coke, Julie Cook, Amy Nichols, Liza McCann, Ian Roberts, Eileen Baildam, Louise Hanna, Olivia Lloyd, Susan Wadeson, Michelle Andrews, Olivia Lloyd, Jane Roach, Phil Riley, Ann McGovern, Verna Cuthbert, Clive Ryder, Janis Scott, Beverley Thomas, Taunton Southwood, Eslam Al-Abadi, Ruth Howman, Sue Wyatt, Gillian Jackson, Mark Wood, Tania Amin, Vanessa VanRooyen, Deborah Burton, Louise Turner, Heather Rostron, Sarah Hanson, Joyce Davidson, Janet Gardner-Medwin, Neil Martin, Sue Ferguson, Liz Waxman, Michael Browne, Roisin Boyle, Emily Blyth, Susanne Cathcart, Mark Friswell, Helen Foster, Alison Swift, Sharmila Jandial, Vicky Stevenson, Debbie Wade, Ethan Sen, Eve Smith, Lisa Qiao, Stuart Watson, Claire Duong, Stephen Crulley, Andrew Davies, Miss Caroline Miller, Lynne Bell, Flora McErlane, Sunil Sampath, Josh Bennet, Sharon King, Helen Venning, Rangaraj Satyapal, Elizabeth Stretton, Mary Jordan, Ellen Mosley, Anna Frost, Lindsay Crate, Kishore Warrier, Stefanie Stafford, Brogan Wrest, Chia-Ping Chou, Paul Pryce, Lucy Wedderburn, Clarissa Pilkington, Nathan Hasson, Muthana Al-Obadi, Giulia Varnier, Sandrine Lacassagne, Sue Maillard, Lauren Stone, Elizabeth Halkon, Virginia Brown, Audrey Juggins, Sally Smith, Sian Lunt, Elli Enayat, Hemlata Varsani, Laura Kassoumeri, Miss Laura Beard, Katie Arnold, Yvonne Glackin, Stephanie Simou, Beverley Almeida, Kiran Nistala, Raquel Marques, Claire Deakin, Parichat Khaosut, Stefanie Dowle, Charalampia Papadopoulou, Shireena Yasin, Christina Boros, Meredyth Wilkinson, Chris Piper, Cerise Johnson-Moore, Lucy Marshall, Kathryn O’Brien, Emily Robinson, Dominic Igbelina, Polly Livermore, Socrates Varakliotis, Rosie Hamilton, Lucy Nguyen, Dario Cancemi, Kevin Murray, Coziana Ciurtin, John Ioannou, Caitlin Clifford, Linda Suffield, Laura Hennelly, Helen Lee, Sam Leach, Helen Smith, Anne-Marie McMahon, Heather Chisem, Jeanette Hall, Amy Huffenberger, Nick Wilkinson, Emma Inness, Eunice Kendall, David Mayers, Ruth Etherton, Danielle Miller, Kathryn Bailey, Jacqui Clinch, Natalie Fineman, Helen Pluess-Hall, Suzanne Sketchley, Melanie Marsh, Anna Fry, Maisy Dawkins-Lloyd, Mashal Asif, Joyce Davidson, Margaret Connon, Lindsay Vallance, Kirsty Haslam, Charlene Bass-Woodcock, Trudy Booth, Louise Akeroyd, Alice Leahy, Amy Collier, Rebecca Cutts, Emma Macleod, Hans De Graaf, Brian Davidson, Sarah Hartfree, Elizabeth Fofana, Lorena Caruana

**Affiliations:** Department of Life Sciences, University of Bath, Bath, UK; Research and Teaching Department, UCL GOS Institute of Child Health, London, UK; NIHR Biomedical Research Centre at GOSH, Great Ormond Street Hospital, London, UK; Centre for Adolescent Rheumatology Versus Arthritis at UCL UCLH and GOSH, London, UK; Department of Rheumatology, Salford Royal Hospital, Northern Care Alliance NHS Foundation Trust, Manchester Academic Health Science Centre, Salford, UK; Division of Musculoskeletal and Dermatological Sciences, Faculty of Biology, Medicine and Health, The University of Manchester, Manchester, UK; Department of Rheumatology, Salford Royal Hospital, Northern Care Alliance NHS Foundation Trust, Manchester Academic Health Science Centre, Salford, UK; Division of Musculoskeletal and Dermatological Sciences, Faculty of Biology, Medicine and Health, The University of Manchester, Manchester, UK; Division of Population Health, Health Services Research & Primary Care, Faculty of Biology, Medicine and Health, The University of Manchester, Manchester, UK; Environmental Autoimmunity Group, Clinical Research Branch, National Institute of Environmental Health Sciences, National Institutes of Health, Bethesda, MD, USA; Muscle Disease Unit, National Institute of Arthritis and Musculoskeletal and Skin Diseases, Bethesda, MD, USA; Department of Medicine, Division of Rheumatology, Johns Hopkins University, Baltimore, MD, USA; Department of Medicine, Division of Rheumatology, Johns Hopkins University, Baltimore, MD, USA; Department of Life Sciences, University of Bath, Bath, UK; Department of Life Sciences, University of Bath, Bath, UK; Royal National Hospital for Rheumatic Diseases Bath NHS Foundation Trust, Bath, UK

**Keywords:** myositis, cancer, autoantibodies, dermatomyositis, Juvenile dermatomyositis

## Abstract

**Objectives:**

Anti-transcriptional intermediary factor 1γ (TIF1γ) autoantibodies are associated with malignancy in adult-onset idiopathic inflammatory myopathy (IIM) and this risk is attenuated if patients are also positive for anti-specificity protein 4 (Sp4) or anti-cell division cycle apoptosis regulator protein 1 (CCAR1). In anti-TIF1γ positive dermatomyositis (DM) patients from the USA, anti-Sp4 and anti-CCAR1 autoantibody frequencies are reported as 32% and 43% in adults and 9% and 19% in juveniles, respectively. This study aims to identify the frequency of anti-Sp4 and anti-CCAR1 in adult and juvenile UK anti-TIF1γ-positive myositis populations and report clinical associations.

**Methods:**

Serum samples from 51 UK participants with adult-onset IIM and 55 UK participants with JDM, all anti-TIF1γ autoantibody positive, and 24 healthy control samples were screened for anti-Sp4 and anti-CCAR1 autoantibodies by ELISA.

**Results:**

In UK adult anti-TIF1γ positive IIM patients, anti-Sp4 and anti-CCAR1 frequencies were 4% (2/51) and 16% (8/51). Both adult patients with anti-Sp4 were also positive for anti-CCAR1. In UK juveniles, anti-Sp4 was not detected and 13% (7/55) had anti-CCAR1 autoantibodies. Nineteen (37%) anti-TIF1γ positive UK adult myositis patients had cancer; neither of the two patients with anti-Sp4 autoantibodies and 25% (2/8) of anti-CCAR1 autoantibody-positive patients had cancer. No anti-Sp4 or anti-CCAR1 clinical associations were identified.

**Conclusion:**

Anti-Sp4 and anti-CCAR1 autoantibodies are less common in the adult UK anti-TIF1γ-positive myositis population compared with published data from the USA, limiting their use as biomarkers for cancer risk. In patients with juvenile onset disease, anti-Sp4 is less frequent in UK patients compared with the USA, but the prevalence of anti-CCAR1 autoantibodies is similar.

Rheumatology key messages

- Anti-Sp4/anti-CCAR1 autoantibodies are rarer in UK anti-TIF1γ-positive adults compared with a similar US patient cohort.
- Anti-Sp4 autoantibodies are rarer in UK anti-TIF1γ-positive children compared with a similar US patient cohort.
- The low prevalence of anti-Sp4 and anti-CCAR1 in UK patients limits their utility as cancer biomarkers.

## Introduction

Autoantibodies are a hallmark of IIM and are important biomarkers allowing patients to be sub-grouped, as they are associated with specific clinical phenotypes and risk factors. Autoantibodies targeting the E3 ubiquitin-ligase family member transcriptional intermediary factor 1γ (TIF1γ), are associated with a significantly increased risk of cancer-associated myositis (CAM) in adults [1], with malignancy rates of 38–80% reported in anti-TIF1γ positive adult dermatomyositis (DM) patients [2]. Recently published International Myositis Assessment and Clinical Studies Group cancer screening guidelines include anti-TIF1γ autoantibodies as a 'high risk' factor for malignancy [3]. Interestingly, anti-TIF1γ is the most common autoantibody seen in juvenile-onset IIM cohorts from the UK and USA, where it is not associated with malignancy but is linked to more severe skin disease and disease chronicity [4–7].

Autoantibodies targeting Sp4 and CCAR1 have recently been identified in IIM patients and have been shown to predominantly occur in DM patients with anti-TIF1γ autoantibodies [8, 9]. Hosono *et al.* reported an anti-Sp4 frequency of 43% in adult DM patients with anti-TIF1γ autoantibodies [8]. None of the anti-TIF1γ autoantibody positive DM patients who were also positive for anti-Sp4 autoantibodies developed malignancy, as compared with 14% of the anti-Sp4 negative anti-TIF1γ autoantibody positive DM patients who developed CAM [8]. Anti-CCAR1 autoantibody frequency in adult DM patients positive for anti-TIF1γ autoantibodies is reported as 30–34% in two cohorts from the USA tested by ELISA [10]. Cancer rates in anti-CCAR1 positive anti-TIF1γ autoantibody positive patients were also significantly lower than in anti-CCAR1 negative anti-TIF1γ positive patients in both tested cohorts from the USA [10]. Given the significantly reduced rate of cancer in anti-TIF1γ positive DM patients that also have anti-Sp4 or anti-CCAR1 autoantibodies, it has been suggested these autoantibodies could prove highly useful biomarkers to refine cancer risk and the need for further investigation and screening in this high-risk population [8–10].

In those with juvenile-onset disease, where an association between anti-TIF1γ and malignancy is not seen, both autoantibodies have been shown to have a lower prevalence: anti-Sp4 autoantibodies were found in 20% of anti-TIF1γ positive juvenile IIM patients from the USA and were associated with Raynaud’s, milder muscle weakness and lower peak aspartate aminotransferase (AST) [11]. Anti-CCAR1 autoantibody frequency was 9% of an anti-TIF1γ positive US juvenile dermatomyositis (JDM) cohort screened by ELISA, and was associated with a lower frequency of cutaneous ulceration and similar clinical features to anti-Sp4 autoantibodies [12].

We aimed to determine the frequency of anti-Sp4 and anti-CCAR1 in UK anti-TIF1γ-positive myositis populations and report any observed clinical associations, including cancer risk.

## Methods

### Patients

Nine hundred and ninety-six adult-onset probable or definite polymyositis (PM)/DM recruited to the UK Myositis Network (UKMYONET) and 381 UK JDM samples recruited to the UK Juvenile Dermatomyositis Cohort and Biomarker Study (JDCBS) [4, 13] were included. Cancer data within UKMYONET were obtained from clinical records and linkage to NHS England’s cancer register.

An additional 11 anti-TIF1γ positive JDM patient samples from the USA were also available to us for analysis. These patients had probable or definite JDM or juvenile polymyositis (JPM) enrolled in the National Institutes of Health’s investigational review board-approved natural history protocols [5].

The earliest serum/plasma samples available were analysed; however, due to variation in when patients were recruited, this ranged widely from disease onset to several years later.

### Anti-TIF1γ autoantibody detection

Protein immunoprecipitation (IP) of radiolabelled K562 cells had previously been performed on all samples to determine the presence of autoantibodies as described [14–17]. In all patients with a compatible 155/140 kDa doublet (*n* = 55 adult IIM and *n* = 65 juvenile DM), anti-TIF1γ ELISA (MBL, RG-7854R) was performed as per the manufacturer’s instructions to confirm the presence of anti-TIF1γ. To remain consistent with previously published data, an ELISA cut-off of >7 units was used [18]. Fifty-one adult and 55 juvenile IIM patient samples were positive by both IP and MLB ELISA.

### Anti-Sp4 autoantibody detection

Anti-Sp4 ELISA was performed as previously described by Hosono *et al*. [8]. Each ELISA plate was calibrated and arbitrary units (AU) calculated using a serial dilution calibration curve of a myositis positive sample. The positive cut off was set as 5 SD above the mean of 24 healthy controls. Anti-GST ELISA was performed on positive samples as described [8] in order to exclude cross reactivity with the recombinant protein GST tag.

### Anti-CCAR1 autoantibody detection

Anti-CCAR1 autoantibody ELISA was performed as previously described by Fiorentino *et al*. [10]. The recombinant CCAR1 C-terminal fragment used in the ELISA was generously gifted to us by Dr Livia Casciola-Rosen.

### Analysis

Categorical variables are reported as percentage and absolute frequencies, and Fisher’s exact test was used to compare groups. Continuous variables are reported as median [interquartile range (IQR)] and Wilcoxon’s rank-sum test was used to compare groups. GraphPad Prism 9.5.1 (GraphPad Software, Boston, MA, USA) was used to perform analyses.

### Ethics approval

Informed written consent to participate in this study was given by all participants; parental consent was given for children in accordance with the Declaration of Helsinki. Participants were recruited across multiple centres and all centres obtained specific ethical approval from their local ethics committees for this study.

## Results

### Anti-TIF1γ autoantibody positive myositis

From the UK adult myositis cohort, 51 (5%) patients were anti-TIF1γ autoantibody positive, 88% White, 84% DM, 80% female, and median (IQR) age at onset 49.3 (37.5–62.7) years (Supplementary Table S1A, available at *Rheumatology* online). Nineteen of 51 (37.3%) of the anti-TIF1γ adult myositis patients had CAM, with seven cases of breast cancer, three ovarian, two lymphoma, and single cases of bowel, hepatic, leukaemia, lung, melanoma, myeloma and oesophageal cancer reported. From the UK JDM myositis cohort, 55 (14%) were anti-TIF1γ positive, 79% White, 58% Female, median age at onset (IQR) 6.9 (3.8–10.1) years (Supplementary Table S1B, available at *Rheumatology* online).

### Anti-Sp4 and anti-CCAR1 frequency

Anti-Sp4 autoantibodies were detected in two (4%) anti-TIF1γ autoantibody positive UK adults and no UK juveniles. Eight (16%) anti-CCAR1 autoantibody positive patients were detected in the anti-TIF1γ positive adult myositis group, two of which were also positive for anti-Sp4. Seven (13%) anti-CCAR1 autoantibody positive patients were detected in the anti-TIF1γ positive UK JDM cohort. No anti-Sp4 or anti-CCAR1 autoantibodies were detected in the 24 healthy controls screened by ELISA.

**Figure 1.**
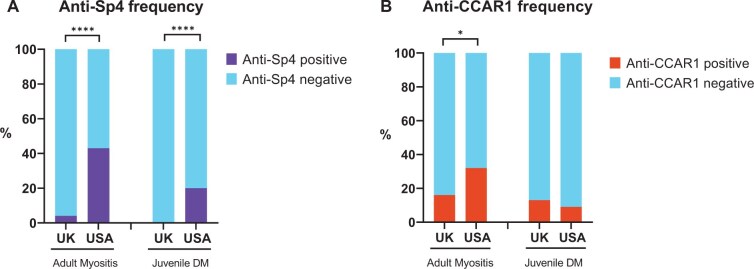
Anti-Sp4 and anti-CCAR1 frequency in anti-TIF1γ positive UK adult and juvenile myositis cohorts compared with previously published data on cohorts from the USA. (**A**) Percentage of anti-Sp4. (**B**) Percentage of anti-CCAR1. US results previously published. References for (**A**): adult myositis [8], juvenile DM [11]. References for (**B**): adult myositis [10], juvenile DM [12]. Antibody frequency compared using Fisher’s exact test, *P ≤ 0.05, ****P ≤0.0001. CCAR1: cell division cycle apoptosis regulator protein 1; DM: dermatomyositis; Sp4: specificity protein 4; TIF1γ: transcriptional intermediary factor 1γ

The frequency of anti-Sp4 and anti-CCAR1 in our UK cohorts compared with previously published data is shown (Fig. 1).

Among 11 anti-TIF1γ positive US JDM patient samples available, anti-TIF1γ positivity was confirmed identically to the UK samples. Sample aliquots from these patients had been independently analysed for anti-CCAR1 and anti-Sp4 in the USA (anti-CCAR1 was tested by Sherman *et al.* [12] and anti-Sp4 by Sherman *et al.* [11]). For anti-Sp4 consistent results were found in all 11 samples. For anti-CCAR1 consistent results were found in 10 samples. The 11th sample was negative on testing in the UK but low positive on testing in the USA.

### Cancer-associated myositis frequency

Neither of the two adults with anti-Sp4 autoantibodies had CAM; however, we could not detect a significant association between anti-Sp4 and cancer frequency due to the very low frequency of anti-Sp4 autoantibody positive patients (Table 1). Two of eight of the anti-CCAR1 autoantibody positive adult myositis patients had cancer, which is similar to the rate of cancer in patients with anti-TIF1γ alone (Table 1).

**Table 1.** Cancer-associated myositis frequency in adult anti-TIF1γ positive IIM patients with anti-CCAR1 and anti-Sp4 autoantibodies

	Anti-Sp4			Anti-CCAR1		
	Negative	Positive	*P-*value	Negative	Positive	*P-*value
CAM frequency, % (*n*/*N*)	39 (19/49)	0 (0/2)	0.5231	39 (17/43)	25 (2/8)	0.6936

Fisher’s exact test used to compare values (GraphPad Prism 9.5.1). CAM: cancer-associated myositis; CCAR1: cell division cycle apoptosis regulator protein 1; IIM: idiopathic inflammatory myopathy; *n*: number; *N*: total number; Sp4: specificity protein 4; TIF1γ: transcriptional intermediary factor 1γ.

### JDM clinical features

Earlier studies have reported increased rates of Raynaud’s, decreased rates of cutaneous ulceration, milder muscle weakness and lower AST levels in JDM patients with anti-TIF1γ-associated autoantibodies [11, 12]. In the UK JDM cohort, there was a slight increased frequency of elevated AST levels in individuals with anti-CCAR1; however, the difference was not significant. No differences in Childhood Myositis Assessment Scale, manual muscle testing 8, Raynaud’s or cutaneous ulceration were observed in the UK JDM individuals with or without anti-CCAR1. Full clinical data was available for 42 of the 55 anti-TIF1γ positive UK JDM participants.

In US JDM patients with anti-Sp4/CCAR1 we observed slightly higher rates of Raynaud’s, no cutaneous ulceration and lower levels of AST compared with anti-TIF1γ positive US patients without anti-TIF1γ-associated autoantibodies; however, the differences were not statistically significant due to the small number of anti-Sp4/CCAR1 positive individuals. Muscle strength/functional measures were not available for the US JDM group.

## Discussion

This is the first description of the prevalence of anti-Sp4 and anti-CCAR1 autoantibodies in a non-US cohort. Unexpectedly, we found the frequency of anti-Sp4 to be much lower in UK myositis populations compared with data published on US cohorts, 4% *vs* 43% in adults [8] and 0% *vs* 20% in children [11]. Anti-CCAR1 was also detected at a lower frequency in UK adults compared with US cohorts, 16% *vs* 32% [10], anti-CCAR1 was found at a similar frequency in UK JDM compared to US JDM, 13% *vs* 9% respectively [12]. Myositis autoantibody prevalence is known to differ between geographically disparate populations, but previously European and US myositis populations have been largely similar, in terms of both autoantibody prevalence and associated clinical phenotypes. We were surprised therefore to observe a much lower frequency of anti-Sp4 and anti-CCAR1 autoantibodies in UK adult anti-TIF1γ positive myositis participants. UK anti-Sp4 positive individuals identified do not have cancer; however, the UK anti-Sp4 positive sample size is very small and therefore no influence on cancer was examined. Similarly, the prevalence of CAM was lower in anti-TIF1γ UK participants with anti-CCAR1 autoantibodies than without these autoantibodies, but low numbers of anti-CCAR1 identified mean that no significant association between anti-CCAR1 autoantibodies and CAM frequency can be confirmed.

The large differences in anti-Sp4 and anti-CCAR1 prevalence between UK and US cohorts are unlikely to be due to inter-laboratory testing variability. To ensure inter-lab comparability we used the same ELISA protocol, developed by Hosono *et al.* [8] and Fiorentino *et al.* [10], and the same recombinant Sp4 and CCAR1 proteins. Additionally, we used the same criteria for defining anti-TIF1γ positivity. A small number of US JDM samples were tested in both UK and US laboratory settings, with one discrepant result for anti-CCAR1. This sample was defined as a low positive on testing in the US lab but negative on testing in the UK. Unspecified differences in testing in different settings could explain some variability in the results but the prevalence of anti-CCAR1 was very similar between UK and US JDM cohorts and only differed in adult patients.

It should be noted that a cut off of 5 SD above the mean of 24 healthy controls was set for the anti-Sp4 positivity in this study and a cut off of 2 SD above the mean of 200 healthy controls was set by Hosono *et al.* [8]. However, this difference in cut offs does not explain the discrepancy between the US and UK cohorts; recalculating UK results with a 2 SD cut off would result in three additional adult IIM anti-Sp4 ‘positives’, two with CAM, and one healthy control anti-Sp4 ‘positive’. We do not consider these true positive as they have significantly lower normalized optical densities (OD) of 0.36–0.26 compared with the true positives classified by the 5 SD cut off of OD 2.70–1.56. Recalculating JDM results with a 2 SD cut off would not result in any additional JDM anti-Sp4 positives.

The reasons behind differing autoantibody prevalence between myositis populations is not well understood. Conceivable explanations include different environmental triggers or subtle differences in study recruitment, such as the timing of sampling in the disease course. The groups tested had similar demographic features and would be expected to have similar genetic backgrounds [19]. Alternatively, the apparent differences may be explained by epitope differences between UK and US populations. Anti-Sp4 was identified using PhIP-Seq [8], and therefore only linear epitopes were identified. Both ELISAs were performed using protein fragments that may not include potential UK specific epitopes.

Due to the small numbers of anti-CCAR1 and anti-Sp4 autoantibodies identified, this study is underpowered to draw firm conclusions on the influence of these autoantibodies on cancer risk and other clinical features. Analysis of other myositis populations is needed to further our understanding of the global prevalence of these autoantibodies and their role in refining disease prognosis, particularly cancer risk.

## Supplementary Material

keae574_Supplementary_Data

## Data Availability

The data underlying this article will be shared on reasonable request to the corresponding author.
