# Supplementary material for: Anti-Sp4 and anti-CCAR1 autoantibodies in UK vs US patients with adult and juvenile-onset anti-TIF1γ-positive myositis
Source: Rheumatology (Oxford). 2024 Nov 7;64(6):3900–5. doi: 10.1093/rheumatology/keae574 (PMC12107081; doi:10.1093/rheumatology/keae574)
Supplement: keae574_Supplementary_Data [file keae574_supplementary_data.docx]

**Supplementary Table S1: Comparing UK and US demographic variables of A: adult and B: juvenile anti-TIF1γ positive myositis patients.**

| **A** | UK IIM | US IIM  Sp4 study (8) | US IIM CCAR1 study (9) | |
| --- | --- | --- | --- | --- |
|  |  |  | Johns Hopkins | Stanford |
| White | 88 (43/49) | 85 (52/61) | 78 (86/110) | 89 (127/142) |
| Female | 80 (41/51) | 85 (52/61) | 80 (88/110) | 82 (117/142) |
| Age at onset | 49.3 (37.5-62.7) | * 46.8 | 51 ± 16 | 45 ± 5 |
| CAM | 37.3 (19/51) | 8 (5/61) | 25 (28/110) | 21 (30/142) |
| DM | 84 (42/50) | 100 (61/61) | 100 (110/110) | 100 (142/142) |

| **B** | UK JM | US JM  Sp4 study (11) | US JM  CCAR1 study (12) |
| --- | --- | --- | --- |
|  |  |  |  |
| White | 79 (31/40) | 79 (89/112) | 77 (116/150) |
| Female | 58 (32/55) | 83 (93/112) | 77 (115/150) |
| Age at onset | 6.9 (3.8-10.1) | * † 7.5 | * 7.14 |
| DM | 100 (55/55) | 92 (104/112) | 100 (150/150) |

Categorical variables are % (number/total number). IIM; idiopathic inflammatory myopathy, JM; juvenile myositis, CAM; cancer associated myositis, DM; dermatomyositis. * Median age of anti-CCAR1 or anti-Sp4 positive and negative groups reported individually in referenced papers, this table includes the mean of these two values. † Age at onset reported for full cohort only, not for anti-TIF1γ positive subgroup.
